# Fate of an Epicure

**Published:** 1888-11

**Authors:** 


### FATE OF AN EPICURE.

Mr. Rogerson, the son of a gentleman of large fortune in Gloucestershire, England, after receiving an excellent education, was sent abroad to make the grand tour. In this journey young Rogerson attended to nothing but the various modes of cookery and the methods of eating and drinking luxuriously. Before his return his father died, when he entered into the possession of a very large fortune and a small landed estate. He was now able to look over his notes of epicurism, and to discover where the most exquisite dishes were to be had and the best books to be procured. He had no other servants in his house but men cooks, for his footman, butler, housekeeper, coachman and grooms were all cooks.

Among those that were more professionally so were three cooks from Italy, one from Florence, another from Sienna, and another from Viterbo, who was employed for the special purpose of dressing one particular dish only—a delicacy of Florence. He had also a German cook for dressing the livers of turkeys. The rest were all French. Besides these he had a messenger constantly traveling between Brittany and London to bring him the eggs of a certain sort of plover, found near St. Malo, and so extravagant was he that a single dinner, though consisting of two dishes only, cost him upward of fifty guineas.

He counted the minutes between his meals, and was wholly absorbed in devising means to indulge his appetite. In the course of nine years he found his table dreadfully abridged by the ruin of his fortune, and himself verging fast to poverty. When he had spent a fortune of £150,000, and was totally ruined, a friend gave him a guinea to keep him from starving; but a short time after he was found dressing an ortolan for himself. Soon afterward he died of starvation.

